# Calibration Procedure of a Multi-Camera System: Process Uncertainty Budget

**DOI:** 10.3390/s23020589

**Published:** 2023-01-04

**Authors:** Ibai Leizea, Imanol Herrera, Pablo Puerto

**Affiliations:** IDEKO, Basque Research and Technology Alliance (BRTA), 20870 Elgoibar, Spain

**Keywords:** multi-camera, measuring, geometry, uncertainty

## Abstract

The Automated six Degrees of Freedom (DoF) definition of industrial components has become an added value in production processes as long as the required accuracy is guaranteed. This is where multi-camera systems are finding their niche in the market. These systems provide, among other things, the ease of automating tracking processes without human intervention and knowledge about vision and/or metrology. In addition, the cost of integrating a new sensor into the complete system is negligible compared to other multi-tracker systems. The increase in information from different points of view in multi-camera systems raises the accuracy, based on the premise that the more points of view, the lower the level of uncertainty. This work is devoted to the calibration procedures of multi-camera systems, which is decisive to achieve high performance, with a particular focus on the uncertainty budget. Moreover, an evaluation methodology has been carried out, which is key to determining the level of accuracy of the measurement system.

## 1. Introduction

Vision systems where six Degrees of Freedom (DoF) positioning is performed by image processing, have experienced a significant growth in recent years in the industrial sector. Although high-precision systems such as laser trackers are already integrated—through norms and standards—in production lines for large-scale measurements, the high economic cost of these devices clearly stand out, among others. A lower cost alternative to laser trackers are the optical CMMs (Coordinate Measuring Machines), also called vision trackers, optical measurement sensors, or even portable CMMs. These portable measuring devices, which have revolutionized the field of vision metrology. They have been included in the initial processes of production lines, in different industrial environments to support the tasks of high precision inspection, tracking and positioning applications, allowing measurements to be taken more quickly and easily. These systems are composed of two or three pre-calibrated cameras, which provide the position of multiple markers.

Moreover, this technology is also increasingly integrating dynamic tracking functionality to better tackle vibrating or non-static environments [1]. Vibrations in the production factories result from a variety of sources such as production machinery, forklifts or crane bridges and they are a common problem for this type of portable device. The degradation in measurement results is given due to the lack of precise positioning of the mechanical structure. Through a self-referencing alternative, which is not dependent on their mounting structure, it is possible to determine the six degrees of freedom of the sensor. This way, it is becoming the alternative especially in automation tasks with robotic arms [2,3]. The Canadian firm Creaform demonstrates the capabilities of its C-Track device in vibration environments, compared to a poly-articulated arm [1]. The result obtained in a non-vibration scenario was 0.011 mm mean square error and a maximum error of 0.031 mm. In a vibration scenario as a square error of 0.013 mm and a maximum error of 0.037 mm was obtained.

Regarding working volumes, most vision trackers are designed for measuring ranges between 1 and 8 m, and in the case of a laser tracker this is even larger. However, as these systems have a single point of view, their working scenarios are limited by the possibility of having parts of the scene unevaluable due to the lack of visibility. Although manufacturers offer the multi-tracking solution, this alternative drives up production costs, not only in the acquisition of more devices but also adapting facilities. Additionally, it considerably lowers the reliability of the solution, considering, for example, the need to reference (calibrate) the devices between them. In addition, even in small work volumes, both have limitations, resulting in a rather difficult task to adapt these systems. It is worse still to use ‘a sledgehammer to crack a nut’.

The human factor dependency as well as the level of complexity in automation is another challenge. The use of a laser tracker implies the need of highly qualified personnel. Vision trackers in turn, require certain knowledge since in most cases are accompanied by a tracking probe, called optical probe systems. The human intervention—based on experience and knowledge—is linked to results, that is, decision-making through subjective criteria by highly trained personnel is one of the key factors in the final accuracy of these systems.

From this perspective, and in view of the difficulty of adapting the measurement scenarios, this is when multi-camera systems are currently gaining ground. These systems consist of a set of cameras strategically located around the working volume. They stand out mainly for their high flexibility and customization provided by having an indefinite number of cameras located in the way that best suits each application. One of the main advantages of multi-camera systems is the capability to achieve higher levels of accuracy through ad hoc system designs for each. The design of these systems is based on determining the number of cameras and the optimal position of each one to maximize overall precision. This adaptability allows one to achieve high levels of precision for 6DoF measurement and/or tracking. The main purpose is to avoid general solutions such as the commercial solutions cited above where the idea is to try to cover as many applications as possible. In addition, the price of including a new camera is negligible compared to adding any of the previous tracker devices. A multi-camera system is an automated solution where the human factor is minimized. It does not require specialized personnel with machine vision or even metrology knowledge. Furthermore, it is a pre-calibrated solution. In the same way that it is not necessary to have knowledge for its use, it is not necessary to have knowledge of calibration procedures. Automated processes also allow reduced times in the tasks of calibration and/or measurement. CMM programs are always executed following the same instructions and report the same results regardless of the user, avoiding measurement uncertainties due to the user.

Commercial companies such as Zeiss or Hexagon also have their niche here (Zeiss AICell and Aicon 3D Arena, respectively). Quality Gate from the Finnish company MapVision or TubeInspect from Aicon, present multi-camera photogrammetric systems with highly linked inspection processes in the automotive sector and in static scenes. OptiTrack, Qualisys or Vicon, among others, are consolidates Motion Capture systems in the market. The company Tecnatom developed the WiiPA system [4] with this technology.

However, existing multi-camera commercial solutions have a scalability limitation, which results in loss of precision, mainly due to calibration processes. As the work area increases, it involves having to design large and high-precision calibration artefacts. This implies non-cost-effective implementation techniques. The most common calibration procedure consists of using known geometric information (e.g., scale bars or patterns) to estimate the transformation—in terms of position and orientation (extrinsic parameters)—between the cameras. These algorithms are well-known as photogrammetric adjustment or bundle adjustment [5,6,7]. The self-calibration process in [8] is supported by a pattern of 13 markers moved to 200 positions and a scale bar of two markers moved to 27 positions. Photogrammetric adjustment is performed in [9]. By positioning a laser pointer in different positions, the projection of these points forms a virtual object (3D point cloud). However, the 3D evaluation is not reliable as it does not have a ground truth to verify. Perez-Cortes et al. [10] also follows a similar strategy, but instead of using a pointer, a sphere is used in 16 positions, and it is solved through a set of camera projections of the epipolar lines. Robson et al. [11,12] carried out a photogrammetric calibration procedure (bundle adjustment) through the Manhattan Vision Metrology System (VMS) pattern to solve intrinsic parameters, extrinsic parameters, and 3D coordinates in one go. As future lines of this work stand out, the evaluation of the multi-camera system using one or more calibrated scale bars in various orientations within the common intersection volume for all the cameras and the evaluation of the performance specifications through the VDI/VDE 2634 [13] are performed. Usamentiaga et al. [14] present a calibration method for a multi-camera system using a 3D object and laser planes, being detected by the multi-camera system. Perez et al. [15] calibrate it using two spheres and Zhang et al. [16] follow planar pattern methodologies to calibrate both intrinsic and extrinsic parameters. Planar pattern calibration techniques where chess boards [17,18,19,20,21,22,23] or other types of 3D patterns [10] are used have limitations in terms of high-range scenarios as very large patterns would be requested and all cameras can see the same work areas. In this sense, contributions such as Xing et al’s. [24] presents multicamera system calibrations with a reduced shared field of view. The intrinsic parameters of these cameras follow the lens model proposed by Luhmann et al. [25].

The widespread acceptance standard ISO 10360-10: 2016 in advanced manufacturing processes makes a laser tracker the measurement tool for high volume industrial metrology applications. The verification of most vision trackers, in turn, is given by the ASME B89.4.22-2004 or DIN EN ISO 10360-2: 2009 standards. These standards are closely linked to robotic and CMM calibrations, always with probing operations, not reporting the accuracy of the measuring device itself. The optical tracking probes entail introducing a new variable—totally dependent on its geometry—into the measurement chain causing greater uncertainty. For example: the Norwegian company Metronor designed long probes to measure interior areas to allow the tracker to continue tracking it [26]. This solution results in designing new external elements to adapt to different circumstances, making it inefficient and imprecise. In multi-camera systems the cameras can measure everything that is visible without the need to design artefacts for it. Few studies have, however, reported precision data or even a vision-system evaluation or verification procedure, according to guidelines like VDI/VDE 2634-part 1 for optical 3D measurement systems. Geodetic Systems, Inc. (GSI) reports precision results for V-STARS/D offering an accuracy of 14 µm + 14 µm/m for V-STARS/D5, 10 µm + 10 µm/m for V-STARS/D12 or 9 µm + 9 µm/m for INCA4. Möller et al. [27] proposed a stereo system consisting of two AICON MoveInspect HR cameras to increase the precision of the absolute position of an industrial machining robot. The location of the robot’s spindle is measured through a specific adapter mounted on the robot’s tool with retro-reflective markers. They report absolute precision up to 50 µm per m^3^ in a range between 1 and 2 m^3^ (conditioned by the markers). It is also concluded that the stereo system can reduce the robot’s absolute positioning error by approximately 0.1 mm compared to a laser-tracker measurement. Since it is a photogrammetry-based system, it depends on several factors, such as camera calibration, marker-detection quality, the image-processing techniques, and resolution. In [8], a study of the uncertainty variables of the tracking of an object in a robotic system is carried out. The number of cameras, positions, angles, size of the object and the type of camera (in terms of sensors) are evaluated in a 4 m^3^ working area. This is compared against a tracker object with a precision of 0.1 mm (2σ) and 0.2 mrad in angular position. In addition, a comparison of the photogrammetric system is carried out with respect to a laser tracker. A multi-camera system of four cameras in a volume of 2 m × 2 m × 1 m. Thus, using a cross-shaped object, a standard deviation error of 0.07 mm is calculated with a maximum error of 0.14 mm. However, a follow-up to the VDI standard is not considered here either. De Cecco et al. [28] present an uncertainty analysis for the reconstruction of a 3D object. Three stages are defined, multi-stereo, multi-camera, and individual stereo. In [19], a theoretical evaluation of the uncertainty analysis is also carried out during a stereo system calibration.

Our study proposes a quantitative evaluation of a multi-camera system based on its calibration procedure through the identification of potential error sources that influence the measurement chain. In this sense, the calibration process is one of the determining factors to achieve high levels of accuracy. Specifically, this work is focused on the influence of intrinsic and extrinsic parameters and the corresponding propagation in the measurement. It follows the idea of applying different calibration strategies in the two-step calibration procedure. Likewise, a measurement system that follows the VDI 2634-part 1 standard to verify the measurement uncertainty.

The presented approach is divided into two main phases. The first section will identify and diagnose the calibration processes involved in the multi-camera system. Whereas the second handles the error budgeting, indicating the factors that are relatively more important. The paper is organized into six sections. In Section 2 the material and methods used in this work are presented. Section 3 handles the calibration experimentation of the multi-camera system. It provides an overview of all the steps carried out for the calibration, as well as the results obtained in this case study together with the identified variables in each phase. Then, Section 4 illustrates the performance of the measurement system through the verification procedure. This analysis is discussed in Section 5 and Section 6 draws the relevant conclusions.

## 2. Materials and Methods

The novelty of this paper lies on the error budgeting to establish the relative weight of each determining source in the different calibration processes. A set of verification experiments are carried out according to the VDI 2634-part 1 standard. This guide guarantees a correct evaluation of photogrammetric systems today.

**Figure 1:**
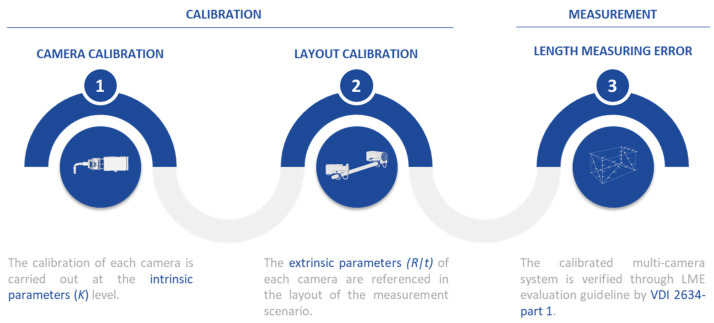
The calibration process of the muti-camera system is divided into two scenarios (camera calibration and measurement). The first scenario (**1**) is concerned with the camera calibration while the second one focuses on referring the layout frame (**2**). Finally, LME evaluation is carried out (**3**).

This work presents the measurement evaluation of a set of calibration methodologies. The process is divided into two main scenarios: calibration and measurement. The calibration scenario provides both the camera calibration itself (Figure 1 (left))—considering the camera as an individual measuring instrument—and the definition of a common reference system (Figure 1 (middle)) that represents the multi-camera system, which is basically the determination of the extrinsic camera parameters (*[R|t]*). In the measurement scenario in turn (Figure 1 (right)), the 3D positioning of a set of markers that follows the geometry suggested by the VDI standard guideline is solved. 

**Figure 2:**
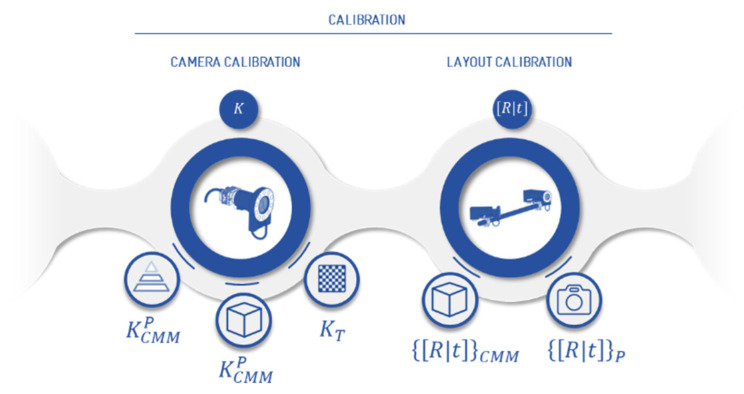
The calibration methodologies studied in this work for the camera calibration (intrinsic camera parameters) are a CMM virtual grid (pyramid/cube) and test-field calibrations, while for the layout calibration a CMM virtual grid (cube) and photogrammetry except for the flat intrinsic calibration pattern and the photogrammetry extrinsic calibration pattern, the rest of the experimental tests are executed in a CMM (ZEISS Prismo 0.9 + L/350 μm). The main goal is to obtain an accurate ground truth in the final verification to determine the error budgeting of the system. More specifically, they have been verified in two measurement scenarios to evaluate the different factors of each calibration process.

This approach analyses two methodologies per each intrinsic and extrinsic calibration process (Figure 2). The intrinsic calibration follows on the one hand, the methodology implemented in [29], where a virtual geometry pattern is optimized to achieve the highest accuracy (Section 3.1.1). On the other hand, a flat pattern composed by retro-reflective targets is photographed in a set of unknown fixed positions (Section 3.1.2). The extrinsic camera calibration, in turn, also follows the virtual grid pattern methodology, but with a different geometry -cube- from the previous one (Section 3.2.1). Moreover, the extrinsic calibration tests are completed with a second strategy of using a 3D pattern set out in the working volume, which is previously measured by a portable photogrammetry system (Section 3.2.2). The output of this system is given by the verification process, where a set of spatial coordinates are measured, again as a virtual grid. The estimation of these results is calculated through the length measuring errors according to LME evaluation guideline by VDI 2634-part 1 [13]. This includes a comparison in terms of length error, between the lengths measured by the photogrammetric system and pre-calibrated scale bars.

**Figure 3:**
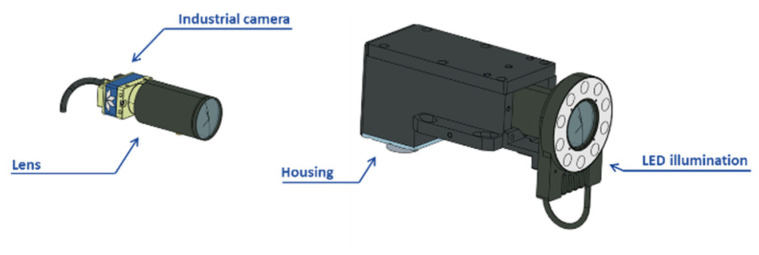

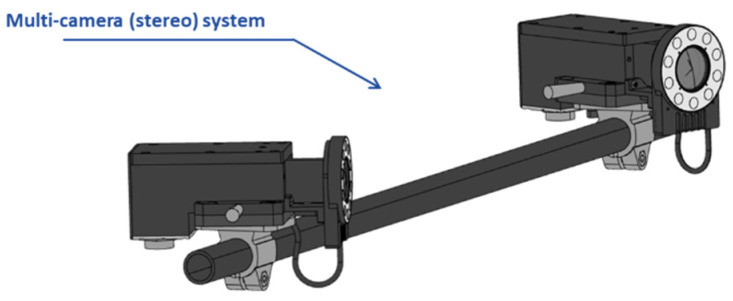
The multi-camera system under study is a stereo-photogrammetric device. Each camera is composed by an industrial camera, lens, and LED illumination. All of this is encapsulated in a housing to avoid noisy environments.

The vision system under study in this paper is a multi-camera system. Specifically, it is a stereo-photogrammetric solution (Figure 3 (down)). The layout is composed by two industrial cameras (Teledyne DALSA Genie Nano 4020, 12.4MP, Schneider Optics APO Xenoplan 2.8 16 mm) individually calibrated in the camera calibration scenario. Afterwards, the results need to be carried into the measurement scenario. In both scenarios, images are taken of reflective non-coded targets. The material property of these elements allows the image detection quality to be the same in both laboratory and industrial scenarios. This is also enabled by the active LED illumination (DCM ALB0810A) integrated by each measuring camera system (Figure 3 (up)). In addition, the camera is encapsulated in a housing manufactured for industrial scenario cases, and thus more efficiently mitigates the effects on the device caused by temperature, humidity, or vibrations. Even so, the tests carried out in this work have been conducted in a controlled laboratory where the above noise factors are mitigated as much as possible.

## 3. Calibration Process

### 3.1. Camera Calibration: Intrinsic Parameters

**Figure 4:**
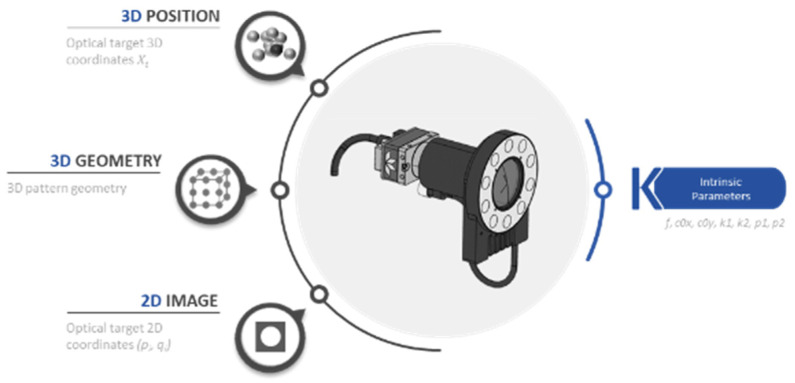
The factors that affect the correct determination of the intrinsic parameters are the 3D position, 3D geometry and 2D image.

The first calibration stage primarily focuses on calibrating the internal camera parameters. Through the optimization of the calibration patterns design, this methodology also allows the camera to be manipulated as an individual measuring instrument. Thus, it can be easily replaced in the measuring system. Through this, it is possible to achieve the maximum level of precision and avoid scalability limitation. The camera calibration consists of calculating the camera focal length and lens distortion parameters (so called intrinsic parameters in machine vision). The 3D coordinates of the pattern, the geometric distribution (position of each marker), and the optical target 2D coordinates are decisive for the calculation of these parameters. As mentioned before, the correct setting for these input variables makes the output intrinsic parameters well determined (Figure 4). A minimum error in this parameter can strongly affect the measurement results.

The widely adopted Brown’s model [30] is used for correcting lens distortions (see Equation (1)).

(1) $$\hat{x}=x+\left(x-c_{0x}\right)\left(k_{1}r^{2}+k_{2}r^{4}\right)+p_{1}\left(r^{2}+2\;{\left(x-c_{0x}\right)}^{2}\right)+2p_{2}\left(x-c_{0x}\right)\left(y-c_{0y}\right)\;\hat{y}=y+\left(y-c_{0y}\right)\left(k_{1}r^{2}+k_{2}r^{4}\right)+p_{2}\left(r^{2}+2\;{\left(y-c_{0y}\right)}^{2}\right)+2p_{1}\left(x-c_{0x}\right)\left(y-c_{0y}\right)$$

where:

- $\left(\hat{x},\hat{y}\right)$ are the corrected point coordinates at the image plane,
- (x,y) are the detected (distorted) point coordinates,
- $\left(c_{0x},c_{0y}\right)$ is the distortion centre,
- $\left(k_{1},k_{2}\right)$ are radial distortion coefficients,
- $\left(p_{1},p_{2}\right)$ are tangential distortion coefficients.
- being $r=\sqrt{{\left(x-c_{0x}\right)}^{2}+{\left(y-c_{0y}\right)}^{2}\;}$

A pre-calibrated 3D pattern and Mendikute et al’s. [29] approach are the chosen strategies among the different alternatives to calibrate the camera parameters. The first consists of a flat pattern that is easy-to-use and allows the instrument to be calibrated in situ. In the second, a virtual grid is adapted to achieve, among other things, a well-conditioned extrinsic parameter and hence less uncertainty. The main drawback of the pattern strategy is the amount of solved extrinsic parameters, which propagates errors. The CMM virtual grid method, in turn, is a high-cost procedure that does not allow one to perform calibration in the measurement scenario itself.

#### 3.1.1. CMM Virtual Grid: Pyramid

**Figure 5:**
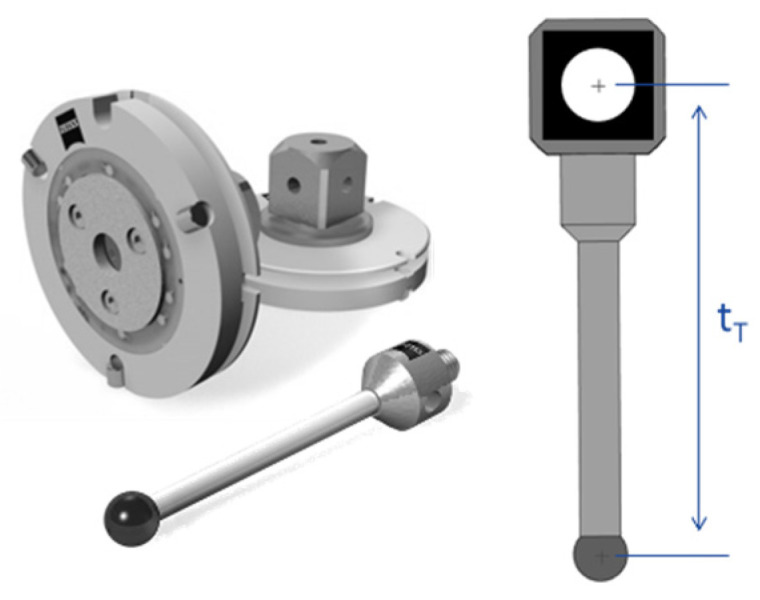
A retroreflective target is placed on a tip, which is previously pre-calibrated to know its 3D position.

As previously mentioned, the idea is to define a virtual grid structure following the process explained in [29], where a single target is captured in different images from different 3D positions (Figure 5). For this work to be self-contained, below is a brief description of this technique.

**Figure 6:**
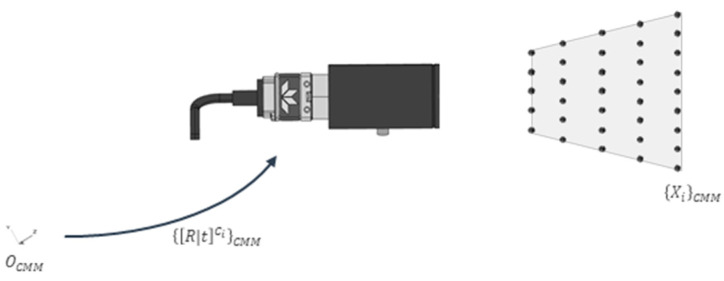
The camera calibration process consists of defining a geometry of 3D coordinates ${\left\{X_{i}\right\}}_{CMM}$ defined in $O_{CMM}$ reference system to compute the intrinsic (K) and extrinsic parameters ${\left\{{\left[R|t\right]}^{C}\right\}}_{CMM}$ of each C camera also defined in the $O_{CMM}$.

A retroreflective target (10 mm diameter) is placed on a previously calibrated probe (in CMM Zeiss O-Inspect). The uncertainty of the movement process is 0.8 µm (1-sigma). The offset obtained here makes it possible to know the 3D position of the target in the CMM coordinate system (Figure 6). This target is placed in certain predefined 3D positions ${\left\{X_{i}\right\}}_{CMM}$ generating a virtual calibration pyramid, where the corresponding image is taken. This pyramid is defined by 10 planes and 10 marker positions in each, a total of 1000 positions.

With all this it is possible to determine the position and orientation ${\left\{{\left[R|t\right]}^{C}\right\}}_{CMM}$ of the *C* camera according to $O_{CMM}$, as well as the internal parameters of the camera ($K_{CMM}$).

The resolution is defined as the non-linear optimization problem solved by the Gauss–Newton method [31] which minimizes the residual vector ${\;||\vec{r}||}^{2}$ norm. The defined calibration geometry is key to have well-conditioned output variables. Hence the need to generate virtual geometries with full freedom. An example of this, is the focal length variable and the extrinsic parameters. The latter is a significant factor due to its propagation in the following calibration stages.

#### 3.1.2. Test-Field Calibration

**Figure 7:**
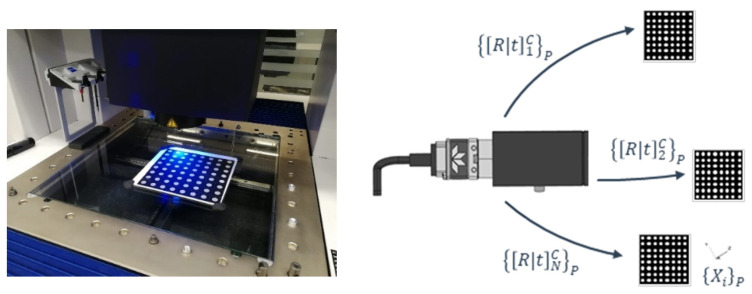
The pre-calibrated pattern (**left**) is located in different positions. In each p position the extrinsic camera parameters are defined and finally, the intrinsic ones are deduced (**right**).

To go through this calibration process, a 64 marker (8 × 8 dots, 140 × 140 mm) ceramic pattern is used (see Figure 7). It is necessary to underline that detection problems were observed in first pilot tests. Some tilt effects were observed in both detection and projection errors using the distortion pattern from Edmund Optics [Optics, E. (s.f.). *Test targets*]. To avoid this problem, the same type of marker as in Section 3.1.1 was selected due to the illumination conditions and to have the same detection uncertainty error in both processes. These circular markers were pre-calibrated in an optical CMM (Zeiss O Inspect), with grid uncertainty below 1 micron.

A calibration test-bench is used for calibrating each camera. A set of images is taken on the calibration grid from different points of view. The imaging configuration is principally designed as [32,33] following the calibration configuration for plane test-fields. However, in this work, although eight positions are proposed by Wester–Ebbinghaus, up to 21 positions are included to cover more areas of the image. Extrinsic parameters are calculated in each of the N images $\left\{{\left[R|t\right]}_{i}\right\},\;i=1\ldots N\;$. Subsequently, along with the processed images, the intrinsic camera parameters ($K_{T}$) are estimated and the extrinsic parameters of each image are refined.

#### 3.1.3. Experimental Evaluation

A repeatability analysis has been performed for each camera. The objective is to evaluate the quality of the calibration and, correspondingly, to assess the accuracy of the integrated predictive models enabling calibration process control.

The experimentation procedure mainly consists of calibrating the two cameras that compose the stereo system. Specifically, the calibration of each of camera is repeated 10 times for both calibration strategies. Table 1 depicts the repeatability of each calibration procedure in terms of intrinsic parameters.

These results not only indicate that a high degree of repeatability is achieved in both processes, but also, the results are definitely similar in all strategies. Attention should be paid to the focal length (f) and distortion centre (cl_0_, rw_0_) variables, with 1 µm and 0.2 pixels of standard deviation (1-sigma), respectively. With these variables it is usually difficult to achieve high levels of repeatability but in this case high precision is achieved regardless of the methodology. The intrinsic calibration performance can also be observed in the resulting reprojection error vector after convergence: 0.06 pixels at *x*-axis and 0.09 pixels at *y*-axis of standard deviation.

### 3.2. Layout Calibration: Extrinsic Parameters

Once the cameras are located on the measurement scenario $O_{L}$, the layout calibration phase is carried out, that is, the extrinsic parameters are solved. As intrinsic phase, here also two types of extrinsic resolution strategies are performed. The first one through a known 3D pattern previously measured with a portable photogrammetric system, and the second one, follows the same procedure as the previous section, but with a cube-based geometry.

It should be noted that, as in the intrinsic calibration scenario, the measurement geometry for the extrinsic calculation is different for each strategy and, moreover, the typology of markers is somewhat different between both of them.

**Figure 8:**
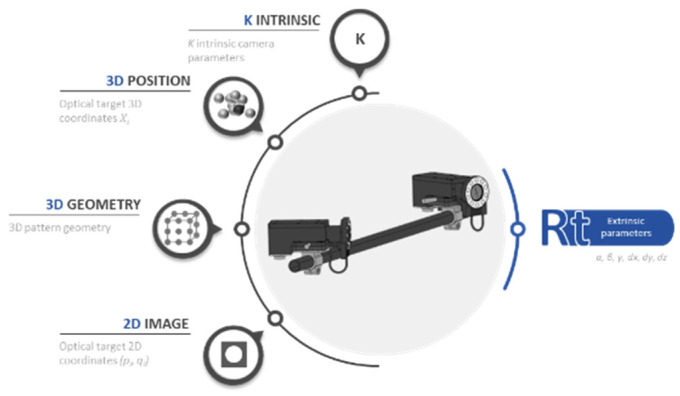
The factors that affect the correct determination of the intrinsic parameters are the 3D position, 3D geometry, 2D image and the K intrinsic parameters.

In addition to the uncertainty resolution of the intrinsic parameters of each camera in the previous phase, it is worth including the 3D position uncertainty of each marker, the 3D geometry that composes the pattern and the 2D uncertainty detection of each marker as input factors. The output of this computation will be the extrinsic parameters represented by *alpha*, *beta*, *gamma* for orientation and *x*,*y*,*z* for translation for each camera that composed the layout (Figure 8).

#### 3.2.1. CMM Virtual Grid: Cube

**Figure 9:**
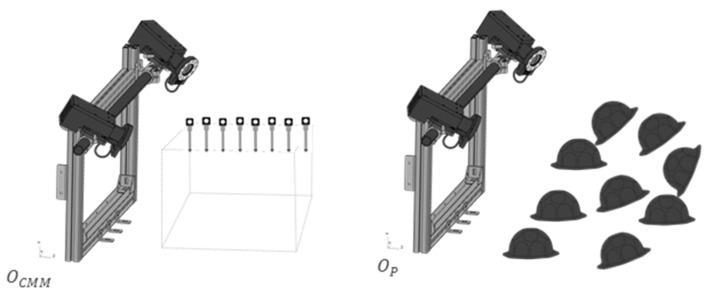
Virtual cube grid (**left**). A single retroreflective target is captured in different images from different points of view. Portable photogrammetry system (**right**). A 3D geometry pattern is resolved by a portable photogrammetry system.

This methodology follows the same steps as Section 3.1.1 with the only difference that instead of using a pyramid, a virtual cube is created that covers the entire working area (see Figure 9 left). In such a way that certain markers will be seen by one or two cameras. It is a 1000-point grid divided into 10 planes, where one of the cameras observes 775 and the other 774 with similar spatial distribution. This difference corresponds to the mechanical assembly error.

The output defines the extrinsic parameters ${\left\{{\left[R|t\right]}^{C}\right\}}_{CMM}$ of each camera in the CMM reference system, that is, the same reference as measurement scenario.

In addition, since the same information is obtained, a calibration of the intrinsic parameters ($K_{CMM}$) is also carried out. This leads to the study of the correlation between both calibration processes since the extrinsic parameters are discarded with previous methods. Therefore, since two types of intrinsic virtual grid calibrations are available in the CMM, from now on both pyramid and cube calibrations will be distinguished as $K_{CMM}^{P}$ and $K_{CMM}^{C}$, respectively. In addition, repeatability values that complement A repeatability analysis has been performed for each camera. The objective is to evaluate the quality of the calibration and, correspondingly, to assess the accuracy of the integrated predictive models enabling calibration process control.

The experimentation procedure mainly consists of calibrating the two cameras that compose the stereo system. Specifically, the calibration of each of camera is repeated 10 times for both calibration strategies. Table 2 depicts the repeatability of each calibration procedure in terms of intrinsic parameters are shown below.

It can be concluded that the repeatability results for this $K_{CMM}^{C}$ are at the same level of those calculated for the $K_{CMM}^{P}$ case.

#### 3.2.2. Photogrammetry

The calibration procedure of this methodology mainly consists of defining a pattern and resolving its 3D geometry by a photogrammetric measurement (Figure 9 right). Therefore, the extrinsic resolution is performed by taking an image for each camera to the resulting 3D scene. The grid is composed of 400 markers, of which 37 and 43 are observed, respectively. 

Since an external device is used to solve the scene, the multi-camera system refers to the zero of the corresponding photogrammetry system ${\left\{{\left[R|t\right]}^{C}\right\}}_{P}$.

#### 3.2.3. Experimental Evaluation

As in the intrinsic scenario, here the precision of the process is also studied (Table 3). The experimentation consists of the calibration of the extrinsic parameters of both cameras using the virtual grid and the 3D pattern obtained by an external photogrammetry system. In particular, the experiment is repeated 10 times to evaluate the repeatability. The input of both methodologies also experimented with each of the two calibrations resulting from the intrinsic process. In addition, for the photogrammetric extrinsic resolution, the cube-shaped intrinsic resolution is also included.

The obtained results served to confirm that the repeatability (1-sigma) of the process does not differ significantly depending on the input. The repeatability of rotation angles is 1 × 10^−5^ and the translation precision in turn, is 1 × 10^−2^ mm for both cases. The error projection ranges between 0.1 and 6 pixels for grid and photogrammetry techniques, respectively.

It should be noted that with photogrammetry strategy there is a slight difference in rotation results—being 1 × 10^−4^ radians—for one of the cameras. This is mainly due to their orientation (less markers are observed), and to the fact that the error is stressed since the 3D geometry is not homogeneous compared to the virtual grid.

## 4. Verification Process

This section explains the metrological assessment as well as the results of the outlined verification procedures. As previously described, this task is carried out in the Zeiss© Prismo CMM which can achieve up to 1 microns of accuracy. Moreover, since it is a small measurement vision system, it is possible to validate an intermediate verification to know the level of accuracy of the multi-camera system.

In this sense, the verification methodology developed in this work for the vision system consists of resolving the quality parameter Length Measuring Error (LME). It is, therefore, the measurement of a point in the three-dimensional space, by knowing the projection of the calibrated cameras with known extrinsic parameters. This problem is called *triangulation*. The detection of the target in both images is required to geometrically determine the target coordinate. In this case, there are three parameters to solve,

(2) $$\theta_{x}=X={\left[x\;y\;z\right]}^{T}$$

where X is the 3D coordinates of the target defined in the same measuring frame at which the camera extrinsic parameters are known $R_{k}$ and $t_{k}$ *k = 1…K* cameras. Each target 3D coordinate can be expressed as $U_{k}={\left[u_{k}{\;v}_{k}{\;w}_{k}\right]}^{T}$ in each camera frame depending on its extrinsic parameters $R_{k}$ and $t_{k}$ as:

(3) $$U_{k}={\;R}_{k}X+t_{k}$$

For each camera, the 3D coordinate $U_{k}$ can be projected into the corresponding camera 2D image plane as $p_{k}$ and $q_{k}$ coordinates, following the widely assumed pin-hole conic projection model in machine vision [34]. This solution can be solved through a non-linear approximation as previously cited. Thus, the partial derivative of an optical target projected on the image with respect to its spatial coordinates is formulated as follows as:

(4) $$J_{x_{k}}={\left(X_{k}\right)}_{2\times 3}=D_{P}D_{U_{X}}$$

where *D_P_* is defined as

(5) $$D_{P}=\left[\begin{array}{ccc} \frac{1}{w_{k}} & 0 & -\frac{u_{k}}{w_{k}^{2}}\\ 0 & \frac{1}{w_{k}} & -\frac{v_{k}}{w_{k}^{2}} \end{array}\right]$$

and $D_{U_{X}}$ expresses the partial derivatives of $U_{k}$ target coordinate at the *k*th camera frame with respect to its X coordinates at the common measuring frame as

(6) $$D_{U_{X}}=R_{j}$$

where $R_{k}$ is the rotation matrix corresponding to the *k*th camera frame.

The following is a more detailed explanation of both verification evaluations based on the results through the described mathematical assumptions.

**Figure 10:**
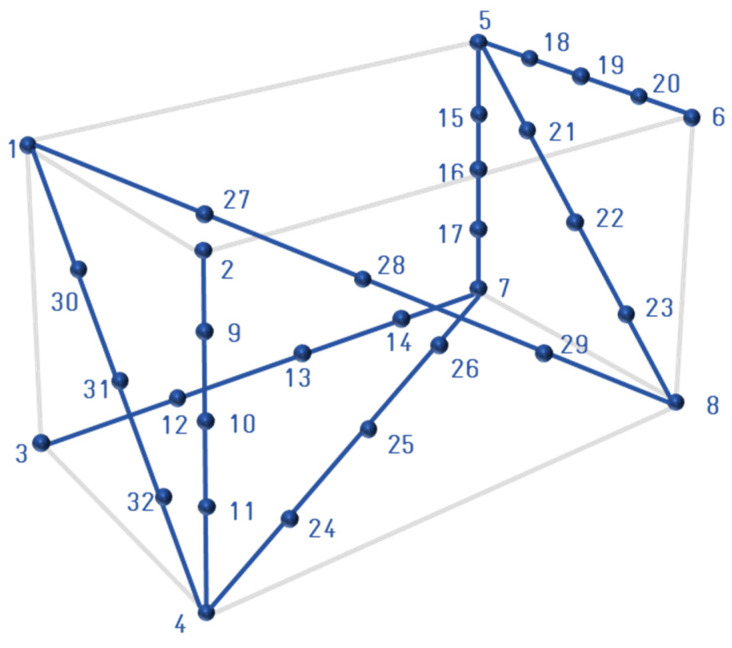
Positions of the bar to verify the accuracy of Optical 3D measuring systems following VDI-VDE 2634.

After inquiring about the standards for the accuracy of metrological vision systems using multiple cameras, it can be said that the VDI-VDE 2634 guideline is the most relevant. This standard consists of three parts from which the first, named Optical 3D measuring systems Imaging systems with point-by-point probing, was selected as it describes how multi-camera systems work. The description of this standard defines how to put the validation bars and the description of the bars themselves (Figure 10).

The positions of the bar are limited by a cube, which is defined by the range of the system. This cube, in turn, defines the length for the bars. The standard is modified to virtually generate the bar using a CMM (Figure 10). It is composed of 32 points for a working area of 215, 320 and 292 mm in x, y, and z of the CMM axis, respectively.

### Experimental Evaluation

Following the above experimentation, the measurement is repeated 10 times for all the combinations of both intrinsic—$K_{CMM}^{P}$ (pyramid)_,_ $K_{CMM}^{C}$ (cube) and $K_{T}$ (test-field)—and extrinsic—${\left\{\left[R|t\right]\right\}}_{CMM}$ (cube) and ${\left\{\left[R|t\right]\right\}}_{P}$ (photogrammetry)—calibrations.

The virtual cube coordinates are compared against the measurements of a multi-camera system with the results shown in Table 4. These results are the maximum, average and standard deviation of the error distance between the ground truth (CMM) and the multi-camera system in Cartesian coordinates.

In view of the results achieved, we can conclude that if higher levels of accuracy are to be obtained, it is necessary to follow a CMM strategy in terms of extrinsic calibration. Similarly, the test-field strategy has a lower performance. In CMM in turn, there is no clear evidence that any factor (K, RT) has a significant determination in the final measurement. All maximum LME are around 30 µm. These results also indicate that there is no clear correlation between the two calibration procedures, although the combination of both calibrations with virtual cube grid offers a slightly better performance, since they are better coupled along with the propagation of the covariance in the calibration chain.

However, it is necessary to pay attention to the $K_{T}\;$and ${\left\{\left[R|t\right]\right\}}_{P}$. Specifically, the measurement data are analysed in detail considering the combination of all the calibrations of this strategy. As Table 5 depicts, it can be concluded that there is a considerable influence of the extrinsic parameters on the final measure. This is largely due to the calibration procedure of the photogrammetric system. More precisely, the chosen geometry causes occlusions which results in a different number of detected markers in each photogrammetric calibration. This effect does not occur, for example, in the case of the CMM, where all the markers are always detected (it is a virtual grid).

Thus, to evaluate the effect of the extrinsic and intrinsic parameters on the results, a swap of calibration is performed, considering the best and worst results. In this case, the 3rd and the 10th measures are selected. It is confirmed (see Table 6) that the extrinsic variability has the higher effect, making it possible to reach CMM precision level or, conversely, definitely negative results.

## 5. Discussion

**Figure 11:**
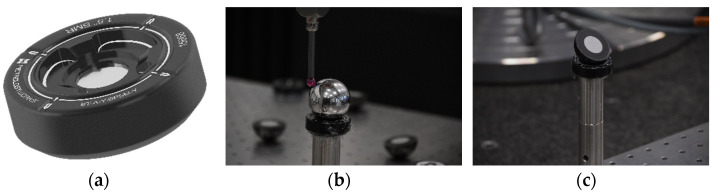
SMR (**a**) as an element in order to have a common reference geometry for calibration with the CMM through a sphere (**b**) or the photogrammetry through a split bearing with a retro reflective marker (**c**).

In view of the results obtained in the previous section, we can conclude that, according to the obtained accuracy, the extrinsic parameters are key in the final measurement result. However, beyond that, it is necessary to emphasize that in order to affirm the above, the calibration geometry of both extrinsic calibration strategies must be identical. Otherwise, differences may arise between the extrinsic calibration between the CMM and portable photogrammetry strategies, which is indeed the current situation. In short, geometry is another key factor to take into account in the extrinsic calibration process. Thus, to be impartial for both cases, a common geometry is defined. For this purpose, a set of Spherically Mounted Retroreflectors (SMRs), commonly known as Nests (see Figure 11a), are distributed along the scene to establish a common centre using tools that can be subsequently measured by CMM and photogrammetry.

The measurement process consists of probing a 1.5″ (38.1 mm) stainless-steel sphere on the CMM, defining its centre (see Figure 11b), and then swapping it with a 1.5″ (38.1 mm) Split Bearing Retro-reflective (SBR), detectable by the photogrammetry system (see Figure 11c). This way, it would be possible to define a common and comparable nominal centre between both metrological tools.

From Table 7 it is possible to conclude that regardless of the results obtained in terms of repeatability, there are no meaningful differences between the two methodologies. Moreover, if a second photogrammetric calibration is included, it is essential to perform an accurate calibration to achieve the same level as the CMM.

Following the analysis, and once it has been confirmed that geometry is a determining factor, it is necessary to focus research on the intrinsic calibration process. It is clear that the first results obtained together with the latter, the $K_{T}\;$calibration, is the one that returns the worst result in the final measurement. This is mainly since the chosen intrinsic calibration methodology has limitations in large scale scenarios. The number of images to be taken increases as the scenario becomes bigger. In addition, to cover the entire scenario, it is necessary to manage the different depths with patterns of different sizes in the same process. All this involved incurring more errors.

Similarly, the CMM methodology also has a drawback in large scenarios. Mainly as it is unfeasible to calibrate the sensors in CMM as the scenarios become larger. CMMs of such a size are not commonly available and the procedure is inefficient. Therefore, a new alternative is proposed to calibrate the intrinsic camera parameters focused on large scenarios through portable photogrammetry. Through this technique, in addition to resolving the 3D coordinates, the intrinsic parameters are also found, as it is a self-calibrating system. These parameters will therefore be subsequently applied as inputs to the multi-camera system.

It can be observed through Table 8 that the combination of both intrinsic and extrinsic parameters with photogrammetry achieve the best results. The conclusion is that both parameters are coupled in the same calibration process.

## 6. Conclusions/Future Work

Vision systems where 6DoF positioning is performed by image processing have become a real alternative to laser trackers in the industrial sector. Like Coordinate Measuring Machines, if the position and orientation of an object with respect to a reference through a laser tracker needed to be found, e.g., to calibrate the TCP of an industrial robot, it is necessary to carry out three consecutive measurements of a stable object and a single tracker, or by including a multi-tracker methodology which implies high costs. This has enabled the design, manufacture, and development of ad-hoc multi-camera systems for each application. At present, it is possible to implement plug&play systems avoiding the above issues. In addition, automatic calibration processes have been obtained, reducing manual intervention to a minimum, thus reducing working times and errors in the processes.

However, to date, most applications that have integrated machine vision measurement systems such as multi-camera systems have always had the main goal of ensuring that errors that accumulated along the entire measurement chain did not affect the final measurement. For instance, an application where a multi-camera system was able to correct the 6DoF positioning of a robotic arm, without going beyond what is necessary for that purpose. If the positioning was guaranteed to be sufficient and correct, the application was validated. For this reason, although the advantages of multi-camera systems have long been proved, their real potential has always been overlooked. This means that multi-camera systems have been constrained by the fears of not meeting the requirements, not being used to their full accuracy potential.

Therefore, a characterisation of the error sources involved in vision systems, and particularly those related to multi-camera systems, has been presented in this work. The main goal has been to evaluate the factors that affect the final measurement, to enable performing what is known as error-budgeting of a measurement system. By means of experimental repeatability tests, it has been possible to carry out the corresponding analysis. Among the different points discussed, it is worth highlighting the identification of the highest number of error sources that influence the measurement chain, to determine the accuracy level that can be achieved, and, on the other hand, the degree of influence of each factor. Specifically, this work focuses on the calibration processes and the different techniques used to evaluate the accuracy of the system. In this work, the VDI2634-part 1 guideline is followed as part of the final verification. In this sense, most of competitors use probing or scanning probes to offer the verification results, without evaluating the system as an individual measuring device.

From the presented data it can be concluded that extrinsic parameters calibration is critical if the geometry and its measurement are correctly determined. Geometry is key in determining extrinsic parameters and this incurs fatal errors and high repeatability. If these conditions are fulfilled and, through the different technologies the scene is correctly measured, no significant changes are observed. So, the next step is to correctly define the intrinsic parameters. Moreover, in this case, it can be confirmed that if both the strategies to calculate the intrinsic and extrinsic parameters are the same, the accuracy is higher, mainly due to the fact of the coupling between both variables of the calibration methodologies.

The performed evaluation, thanks to the knowledge of the contribution of each calibration process in the measurement chain, can be enhanced in the future to estimate and model each of the identified factors. This information could be used to develop simulation processes for the preliminary design of calibration and measurement processes. It could also be used to predict the behaviour of the multi-camera system through designing simulations in the calibration and measurement processes.

It would also be possible to assess in more detail each calibration process focusing on the intrinsic ones. An intermediate characterisation of the intrinsic parameters, i.e., to perform intermediate verifications, by means of dimensional verification rules.

Finally, the motivation to implement dynamic reference, as reported in other studies in the state of the art, should be emphasised.

## Figures and Tables

**Table 1:** The precision results (1-sigma) of the intrinsic parameters of each C camera for pyramid virtual grid and test-field calibration.

Camera	Strategy	*f* (mm)	*cl*_0_ (Pixel)	*rw*_0_ (Pixel)	*k*_1_ (Pixel^−2^)	*k*_2_ (Pixel^−4^)	*p*_1_ (Pixel^−1^)	*p*_2_ (Pixel^−1^)	*RMS*	
									*x*	*y*
*C1*	*CMM* *(* $K_{CMM}^{c1}$ *)*	0.0013	0.2445	0.2053	1.53 × 10^−12^	1.95 × 10^−19^	1.45 × 10^−9^	4.89 × 10^−10^	0.066	0.058
	*Test-field (* $K_{T}^{c1}$ *)*	0.001	0.2434	0.1324	4.79 × 10^−12^	6.32 × 10^−19^	1.68 × 10^−9^	1.89 × 10^−9^	0.067	0.09
*C2*	*CMM* *(* $K_{CMM}^{c2}$ *)*	0.0015	0.2899	0.157	1.15 × 10^−12^	1.48 × 10^−19^	8.11 × 10^−10^	1.41 × 10^−9^	0.076	0.09
	*Test-field (* $K_{T}^{c2}$ *)*	0.0011	0.1244	0.1488	4.7 × 10^−12^	4.79 × 10^−19^	1.8 × 10^−9^	1.311 × 10^−9^	0.06	0.089

**Table 2:** The precision results (1-sigma) of the intrinsic parameters of each C camera for cube virtual grid calibration.

Camera	Strategy	*f* (mm)	*cl*_0_ (Pixel)	*rw*_0_ (Pixel)	*k*_1_ (Pixel^−2^)	*k*_2_ (Pixel^−4^)	*p*_1_ (Pixel^−1^)	*p*_2_ (Pixel^−1^)	*RMS*	
									*x*	*y*
*C1*	*CMM* *(* $K_{CMM}^{c1}$ *)*	0.0024	0.2267	0.1241	2.79 × 10^−12^	3.28 × 10^−19^	1.45 × 10^−9^	8.17 × 10^−10^	0.048	0.062
*C2*	*CMM* *(* $K_{CMM}^{c2}$ *)*	0.0012	0.2216	0.1093	2.22 × 10^−12^	3.03 × 10^−19^	9.62 × 10^−10^	5.35 × 10^−10^	0.073	0.095

**Table 3:** The precision results for the extrinsic calibration procedure, considering the combination of different strategies from intrinsic and extrinsic parameters for both cameras.

Strategy	*K* (Input)	Camera	*α* (Rad)	*β* (Rad)	*γ* (Rad)	*dX* (mm)	*dY* (mm)	*dZ* (mm)
${\left\{\left[R|t\right]\right\}}_{CMM}$	$K_{CMM}^{P}$	*C1*	4.12 × 10^−5^	4.37 × 10^−5^	2.08 × 10^−5^	0.042	0.034	0.055
		*C2*	2.83 × 10^−5^	4.03 × 10^−5^	1.36 × 10^−5^	0.056	0.035	0.044
	$K_{T}$	*C1*	3.18 × 10^−5^	4.51 × 10^−5^	1.75 × 10^−5^	0.043	0.023	0.07
		*C2*	3.26 × 10^−5^	2.34 × 10^−5^	1.42 × 10^−5^	0.029	0.036	0.058
${\left\{\left[R|t\right]\right\}}_{P}$	$K_{CMM}^{P}$	*C1*	2.97 × 10^−4^	2.58 × 10^−4^	2.97 × 10^−4^	0.078	0.022	0.071
		*C2*	2.49 × 10^−4^	9.34 × 10^−5^	2.68 × 10^−4^	0.039	0.02	0.075
	$K_{CMM}^{C}$	*C1*	3.16 × 10^−4^	2.26 × 10^−4^	3.03 × 10^−4^	0.071	0.013	0.103
		*C2*	2.47 × 10^−4^	9.75 × 10^−5^	2.59 × 10^−4^	0.045	0.025	0.091
	$K_{T}$	*C1*	6.58 × 10^−4^	3.13 × 10^−4^	2.98 × 10^−4^	0.083	0.068	0.105
		*C2*	5.6 × 10^−4^	2.87 × 10^−4^	3.01 × 10^−4^	0.091	0.079	0.113

**Table 4:** LME maximum, average and standard deviation (k = 1) through VDI guideline for each calibration combination.

*K*	[R|t]	*LME Max* (mm)	*LME µ* (mm)	*LME σ* (mm)
$K_{CMM}^{P}$	${\left\{\left[R|t\right]\right\}}_{CMM}$	0.039	0.018	0.018
$K_{CMM}^{C}$		0.03	0.013	0.008
$K_{T}$		0.051	0.019	0.012
$K_{CMM}^{P}$	${\left\{\left[R|t\right]\right\}}_{P}$	0.108	0.0328	0.022
$K_{CMM}^{C}$		0.103	0.046	0.034
$K_{T}$		0.16	0.036	0.0244

**Table 5:** VDI results for strategy$\;K_{T}\;$ and ${\left\{\left[R|t\right]\right\}}_{P}\;$ in a 10-repetition trial.

*K*	$\left[R|t\right]\;K_{T}{\left\{\left[R|t\right]\right\}}_{P}1$	Repeat	*LME Max* (mm)	*LME µ* (mm)	*LME σ* (mm)
*K_T_*	{[*R*|*t*]}_*P*_	1	0.16	0.001	0.046
		2	0.171	0.0008	0.057
		3	0.315	0.037	0.139
		4	0.172	0.006	0.067
		5	0.249	0.027	0.112
		6	0.143	0.001	0.061
		7	0.199	0.013	0.084
		8	0.196	0.01	0.082
		9	0.141	0.0003	0.049
		10	0.069	0.003	0.024

**Table 6:** The combination of different repeats of intrinsic and extrinsic calibrations.

*K*	[R|t]	*LME Max* (mm)	*LME µ* (mm)	*LME σ* (mm)
$K_{T}{}^{3}$	${\left\{\left[R|t\right]\right\}}_{P}{}^{3}$	0.315	0.037	0.139
$K_{T}{}^{10}$	${\left\{\left[R|t\right]\right\}}_{P}{}^{10}$	0.069	0.003	0.024
$K_{T}{}^{10}$	${\left\{\left[R|t\right]\right\}}_{P}{}^{3}$	0.311	0.037	0.142
$K_{T}{}^{3}$	${\left\{\left[R|t\right]\right\}}_{P}{}^{10}$	0.065	0.001	0.024
$K_{T}{}^{1}$	${\left\{\left[R|t\right]\right\}}_{P}{}^{3}$	0.313	0.037	0.139
$K_{T}{}^{1}$	${\left\{\left[R|t\right]\right\}}_{P}{}^{10}$	0.073	0.003	0.025

**Table 7:** LME for the experimental test where the extrinsic calibration geometry is the same for different calibration processes. In addition, a second extrinsic calibration process using portable photogrammetry is included as a repeatability test.

K	[R|t]	LME Max (mm)	LME µ (mm)	LME σ (mm)
$K_{CMM}^{P}$	${\left\{\left[R|t\right]\right\}}_{CMM}$	0.132	0.013	0.049
$K_{CMM}^{C}$		0.079	0.007	0.032
$K_{T}$		0.231	0.028	0.065
$K_{CMM}^{P}$	${\left\{\left[R|t\right]\right\}}_{P}$	0.114	0.010	0.043
$K_{CMM}^{C}$		0.080	0.007	0.030
$K_{T}$		0.237	0.033	0.077
$K_{CMM}^{P}$	${\left\{\left[R|t\right]\right\}}_{P}$	0.13	0.014	0.048
$K_{CMM}^{C}$		0.082	0.006	0.032
$K_{T}$		0.239	0.029	0.064

**Table 8:** The photogrammetry intrinsic calibration is included within the different calibration techniques.

K	[R|t]	LME Max (mm)	LME µ (mm)	LME σ (mm)
$K_{CMM}^{P}$	${\left\{\left[R|t\right]\right\}}_{CMM}$	0.132	0.013	0.049
$K_{CMM}^{C}$		0.079	0.007	0.032
$K_{T}$		0.231	0.028	0.065
$K_{PH}$		0.114	0.014	0.05
$K_{CMM}^{P}$	${\left\{\left[R|t\right]\right\}}_{P}$	0.114	0.010	0.043
$K_{CMM}^{C}$		0.080	0.007	0.030
$K_{T}$		0.237	0.033	0.077
$K_{PH}$		0.063	0.006	0.031

## Data Availability

Not applicable.

## Abbreviations

DoF	Degrees of Freedom
CMM	Coordinate Measuring Machine
K	Intrinsic/internal camera parameters
[*R*|*t*]	Extrinsic/external camera parameters
